# Robot-assisted total pelvic exenteration for rectal cancer after neoadjuvant chemoradiotherapy: a case report

**DOI:** 10.1186/s40792-022-01547-x

**Published:** 2022-10-07

**Authors:** Kyoichi Kihara, Yuri Koyama, Takehiko Hanaki, Kozo Miyatani, Tomoyuki Matsunaga, Manabu Yamamoto, Shuichi Morizane, Naruo Tokuyasu, Teruhisa Sakamoto, Yoshiyuki Fujiwara

**Affiliations:** 1 Division of Gastrointestinal and Pediatric Surgery, Department of Surgery, Tottori University Faculty of Medicine, 36-1 Nishimachi, Yonago City, Tottori 683-8504 Japan; 2 Division of Urology, Department of Surgery, Tottori University Faculty of Medicine, 36-1 Nishimachi, Yonago City, Tottori 683-8504 Japan

**Keywords:** Pelvic exenterations, Colorectal neoplasms, Robot surgery, Laparoscopy, Total, Chemoradiotherapy, Prostatectomy

## Abstract

**Background:**

There are numerous indications for minimally invasive surgery. However, the laparoscopic approach for extended pelvic surgery is currently provided by only a few institutions specializing in cancer treatment, primarily because of technical difficulties that arise in cases involving a narrow pelvis and rigid forceps. We report a case of robot-assisted total pelvic exenteration for rectal cancer involving the prostate. We assessed the feasibility of robot-assisted total pelvic exenteration and compared the short-term outcomes of other conventional and minimally invasive approaches.

**Case presentation:**

A 67-year-old man was referred to our hospital after positive fecal blood test results. The initial diagnosis was clinical T4bN2aM0, Stage IIIC rectal cancer involving the prostate. The patient underwent neoadjuvant chemoradiotherapy. Consequently, robot-assisted total pelvic exenteration with an ileal conduit and end colostomy creation were performed. The total operative duration was 9 h and 20 min. The durations of robot console usage by the colorectal and urological teams were 2 h 9 min and 2 h 23 min, respectively. The patient was discharged on postoperative day 21. The pathological diagnosis was T4b (prostate) N0M0, Stage IIC. The resection margin was 2.5 mm. During reassessment at 2 years after resection, no evidence of recurrence was observed.

**Conclusions:**

Robot-assisted total pelvic exenteration was performed for a patient with advanced rectal cancer without serious complications. Robot-assisted total pelvic exenteration may provide the advantages of minimally invasive surgery, particularly in the enclosed space of the pelvis.

## Background

Preoperative staging has improved because of enhanced imaging technology and the multidisciplinary approach to rectal cancer have facilitated patient selection [1]; however, some patients require total pelvic exenteration (TPE). TPE involves total surgical removal of the pelvic viscera, including the bladder, rectum, and reproductive organs [2]. Minimally invasive colorectal cancer surgery has become widely accepted. Some institutions specializing in cancer treatment have reported the safety and feasibility of laparoscopic TPE [3, 4]. However, the manipulation of rigid forceps against rectal cancer adherent to adjacent organs within a narrow pelvis remains a complicated and challenging surgical procedure that is regarded as an exclusion criterion for laparoscopic resection at most hospitals [5, 6]. A robotic approach to TPE may be advantageous over conventional laparoscopic surgery because of the enhanced three-dimensional views and stable magnified views, as well as the increased dexterity of EndoWrist^®^ (Intuitive Surgical, Sunnyvale, CA, USA) instruments, which provide a greater range of motion while eliminating tremor. We describe robot-assisted (RA) TPE (RA-TPE) performed for a patient with advanced rectal cancer involving the prostate, the status after neoadjuvant chemoradiotherapy (CRT), and the feasibility RA-TPE compared to laparoscopic TPE, conventional TPE, and simultaneous RA surgery for synchronous primary rectal cancer and prostate cancer.

## Case presentation

**Fig. 1:**
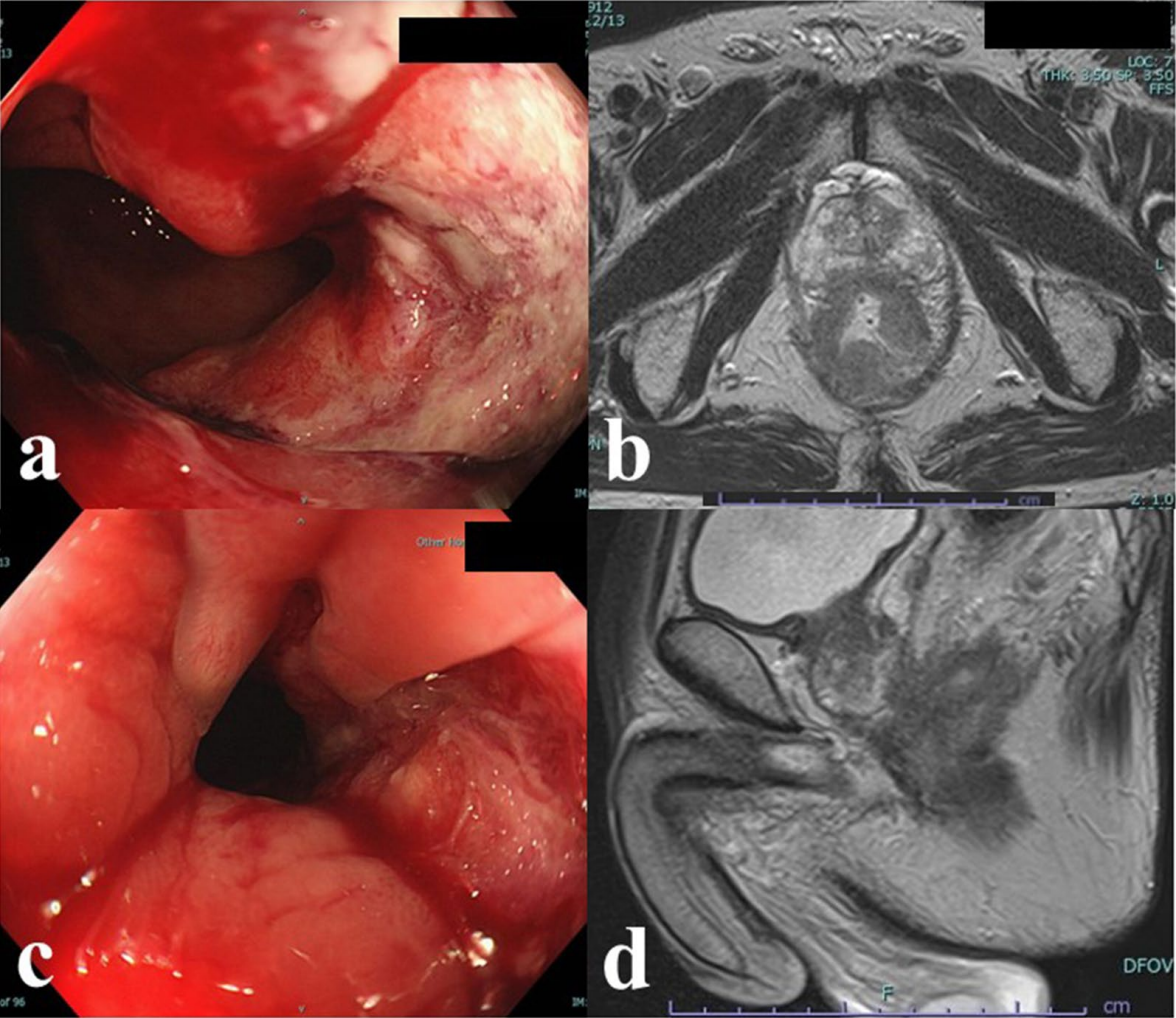
**a** Colonoscopy image and **b** magnetic resonance image at the time of the initial diagnosis. The ulcerative tumor is located in the lower rectum and proctodeum. Biopsy revealed tubular adenocarcinoma. **c** Colonoscopy image and **d** magnetic resonance image after neoadjuvant chemoradiotherapy. Partial response was observed. However, the prostate was still involved

A 67-year-old man was referred to our hospital after positive fecal blood test results. The patient was 164 cm tall and weighed approximately 51 kg. Further examination revealed advanced rectal cancer located below the peritoneal reflection and at the level of the dentate line that involved the prostate (Fig. 1a, b). According to the eighth edition of the TNM classification set by the Union for International Cancer Control, the initial diagnosis was clinical T4b (prostate) N2aM0, stage IIIC without lateral lymph node metastasis.

Neoadjuvant CRT consisting of 45 Gy in 25 fractions combined with tegafur, uracil, and folinic acid was administered. After neoadjuvant CRT, the tumor size decreased from 55 to 45 mm, but the prostate was still involved (Fig. 1c, d). Possible surgical procedures were discussed at a multidisciplinary conference. Partial prostatectomy was thought to be a suboptimal procedure for attaining negative tumor resection margins. The advantages and disadvantages of bladder-sparing prostatectomy with vesicourethral anastomosis were considered. For patients with lower rectal cancer who undergo neoadjuvant CRT, a diverting ileostomy is usually planned to avoid anastomotic leakage and two-stage stoma closure because of the impact of neoadjuvant CRT on anastomoses. However, there is little evidence supporting the feasibility of vesicourethral anastomoses after CRT. Because of the concern regarding refractory vesicourethral leakage in the dead space of the pelvic cavity after TPE, we abandoned vesicourethral anastomosis and pursued RA-TPE. Because our center for minimally invasive surgery had been performing robotic surgeries for more than 10 years, the institutional ethical review board decided that our proposal for RA-TPE successfully met the guidelines for safe introduction of highly complex medical techniques set by the Japanese Ministry of Health, Labor, and Welfare. The expense for this novel surgery was not covered by the Japanese health insurance; therefore, it was covered by our institution.

### Surgical procedure

**Fig. 2:**
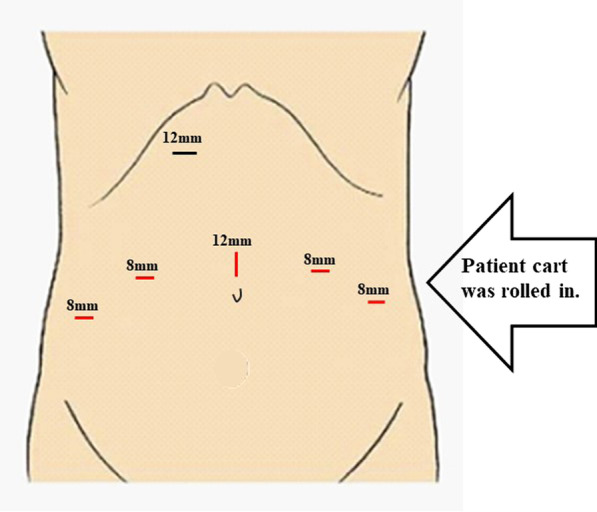
Schema of port placement. A total of five robotic ports were placed (red line), and another conventional laparoscopic 12-mm port (black line) was inserted in the right upper quadrant by an assistant operator. The Da Vinci Xi^®^ patient cart was rolled to the left side of the patient only once during surgery

Five robotic ports were placed, including one 12-mm port (Fig. 2). Another 12-mm conventional laparoscopic port for an assistant operator was placed in the right upper quadrant because of the uncertainties regarding the adequacy of current measures to achieve effective hemostasis using robotic instruments. The patient was positioned in the modified Lloyd–Davies position and tilted in the Trendelenburg position by 17 to 20 degrees. The Da Vinci Xi^®^ (Intuitive Surgical, Sunnyvale, CA, USA) surgical system cart was installed on the left side of the patient. First, the colorectal surgeons targeted the left external iliac artery with the robotic axis for mobilization of the left mesocolon. The inferior mesenteric artery was ligated for total mesorectal excision. After the pelvic phase, the axis changed toward the peritoneal reflection. Mobilization of the posterior mesorectum proceeded down to the pelvic floor. Subsequently, the urological surgeons began operating without repositioning the robot. The bilateral urinary tracts were taped and dissected toward the bladder. The Retzius space was dissected to reach the prostatic apex. The deep dorsal vein of the penis was divided and sealed with a robotic vessel sealer. Santorini’s venous plexus was tied using 3–0 V-Loc^®^ (Medtronic, Minneapolis, MN, USA). Renal damage was minimized by transecting the ureters at the end of the procedure. The RA procedure was completed by amputation of the sigmoid colon using a surgical stapler, and the specimen was retrieved through perineal resection. For patients who have undergone neoadjuvant chemoradiotherapy, prophylactic lymph node dissection of the pelvic wall is not routinely performed at our institution. An ileal conduit and ureteric anastomoses were created extracorporeally with a mini-laparotomy measuring 7 cm.

## Results

**Fig. 3:**
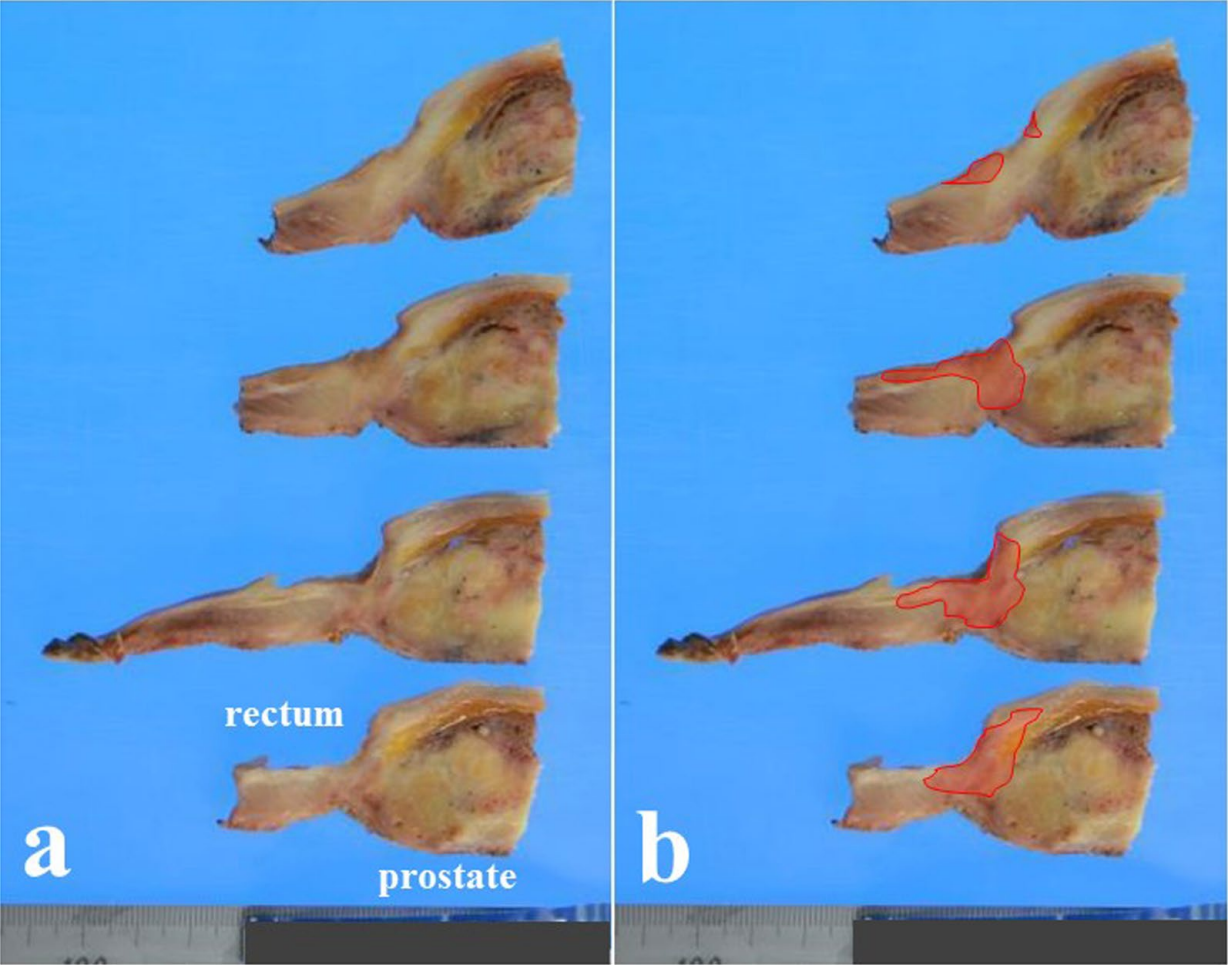
Slices of the resected specimen fixed in formalin. **a** Boundary line between the rectum and prostate was unclear because of tumor cell infiltration. **b** Mapping of viable tumor cells is shown as the area in red. The depth was diagnosed as ypT4b (prostate), and the resection margin was 2.5 mm

The duration of the entire procedure was 9 h and 20 min. The durations of robot console usage by the colorectal and urological teams were 2 h 9 min and 2 h 23 min, respectively. The estimated volume of blood loss was 200 ml. The pathological diagnosis was ypT4b (prostate) N0M0, stage IIC, with a resection margin of 2.5 mm (Fig. 3). Oral intake was reintroduced on postoperative day 3, starting with a liquid diet. Without symptoms, laboratory data indicating mild inflammation were observed and antibiotics were administered until postoperative day 14. The patient had a postoperative hospital stay of 21 days. At 2 years after resection, there was no evidence of cancer recurrence.

## Discussion

Although the global standard of management for advanced rectal cancer is neoadjuvant radiotherapy with or without chemotherapy and total mesorectal excision [7], the Japanese guidelines for the treatment of colorectal cancer recommend total mesorectal excision with lateral lymph node dissection based on evidence from Japanese Clinical Oncology Group 0212 [8, 9]. Prophylactic lateral lymph node dissection contributes to a lower rate of locoregional recurrence, but it does not enhance the overall survival of advanced rectal cancer patients without radiotherapy. To date, there has been only one randomized controlled trial comparing the outcomes of lateral lymph node dissection and nerve-preserving resection for patients with rectal cancer after preoperative radiotherapy; that study consisted of 51 patients and showed no difference in survival or disease-free survival [10]. It remains controversial whether neoadjuvant radiotherapy can be a substitute for prophylactic lateral lymph node dissection. Our institution conforms to the global standard for neoadjuvant radiotherapy without prophylactic lateral lymph node dissection. If locoregional recurrence is detected only in the pelvic wall, then metachronous lateral lymph node dissection of the recurrent side is proposed. During the present study, our surgical decisions considered the expected effects of neoadjuvant radiotherapy, such as tumor size reduction and possible preservation of the prostate.

The first use of RA-TPE was published in 2011 by Vasilescu et al., who performed RA-TPE for recurrent endometrial cancer [11]. Subsequently, Shin et al. reported RA-TPE for rectal cancer in 2014 [12]. Although both RA prostatectomy and RA rectal resection are becoming common choices for malignancies originating from the prostate and rectum, respectively, RA-TPE has been reported for only a few cases. For advanced colorectal cancer, only four case reports of RA-TPE were found by cross-searching “colorectal neoplasms,” “robot surgery,” and “pelvic exenterations” in MEDLINE (Table 1) [12–15]. Including our case, operative times ranged between 200 and 560 min, and the amount of blood loss was 100 to 350 ml (missing in one case). The most severe complication was ureteric stricture requiring stent placement. The length of the postoperative hospital stay ranged from 7 to 21 days. All cases were T4, and there was only one case of stage IV [14]. Our case had the longest follow-up period (24 months). Oncological outcome data of RA-TPE, which are the most meaningful data, are still lacking because of the small number of reported cases to date; therefore, its prevalence remains unknown.

**Table 1:** Previous reported cases of RA-TPE against primary rectal cancer

References (published year)	Age	Sex	BMI	Preoperative radiation	Neoadjuvant chemotherapy	Robot generation^†^	Operative duration (min)	Amount of blood loss (ml)	T^§^	N^§^	M^§^	Stage^§^	RM (mm)	Complications (Clavien–Dindo classification)	Hospital stay after surgery (days)	Recurrence	Follow-up period (months)
Shin JW. (2014) [12]	41	M	18.0	Yes	Yes	NA	480	300	cT4b	N2	M0	cIIIC	NA	None	8	NA	NA
Winters BR. (2015) [13]	61	M	24.0	Yes	Yes (FOLFOXIRI)	Si	570	350	T4b	N2	M0	IIIC	NA	None	7	NA	NA
Wiliams M. (2020) [14]	46	M	18.1	Yes	Yes (mFOLFOX6)	NA	200	NA	T4	N2a	M1	IV	4.0	UTI (II), ureteric stricture (IIIa)	9	Yes (not local)	21
Stefan S (2022) [15]	62	M	32.0	Yes	Yes (capecitabine)	X	400	100	ypT4b	N1b	MX	ypIIIc	> 1.0	ileus (II) and UTI (II)	11	NA	1
Our case	67	M	19.0	Yes	Yes (tegafur/uracil)	Xi	560	200	ypT4b	N0	M0	ypIIc	2.5	unknown fever (II)	21	None	24

RM; resection margin, UTI; urinary tract infection

^†^Generation of Da Vinci Systems^®^ (Intuitive Surgical, Sunnyvale, CA, USA)

^§^According to Union for International Cancer Control TNM classification 8th edition

Heah et al. reported three cases of RA bladder-sparing pelvic exenteration for colorectal cancer [16]. All patients underwent neoadjuvant CRT before resection. In contrast, salvage radical prostatectomy has not been widely accepted as treatment for radiation-recurrent prostate cancer because of the surgical morbidity associated with the procedure. The incidence of anastomotic stricture in salvage radical prostatectomy varies from 9% to 33%, whereas that of urinary continence ranges from 33% to 80% [17]. These results are reflected in the very low prevalence of salvage radical prostatectomy [18]. Considering these risks, vesicourethral anastomosis was avoided in our case, despite the advantages of enhanced dexterity with a robotic anastomosis compared to that of the laparoscopic approach. We believe that further validation during patient selection after CRT is required to estimate the feasibility of vesicourethral anastomosis and RA bladder-sparing pelvic exenteration.

A brief review of the feasibility of RA-TPE performed by comparing the short-term outcomes of the other approaches, including conventional TPE, laparoscopic TPE, simultaneous RA surgery of synchronous primary rectal and prostate cancer, is shown in Table 2. The short-term outcomes of the reported RA-TPE cases seem considerable based on the latest report of a large-scale investigation of TPE [19]. The median operative durations were 480 min (range 200–570 min) for RA-TPE cases and 462 min (range 333–582 min) for conventional TPE. The median amount of blood loss was 250 ml (range 100–350 ml) in the former group, and 50% of the patients who underwent conventional TPE required transfusion. Major complications, classified as Clavien–Dindo grade 3 or greater, were observed in one of five patients of RA-TPE and 120 of 749 patients of conventional TPE. Fukuta et al. reported simultaneous RA resection of synchronous rectal cancer and prostate cancer with vesicourethral anastomosis [20]. Preoperative radiotherapy was not introduced. Four patients underwent total mesorectal excision and radical prostatectomy separately, and only one patient underwent en bloc resection of both cancers. The short-term outcomes, including operative duration, amount of blood loss, and hospital stay, indicated the possible feasibility of simultaneous RA rectal resection and RA radical prostatectomy, even though one patient developed colorectal anastomotic leakage and two patients experienced vesicourethral anastomotic leakage. Their outcomes are quite similar to those of RA-TPE. The operative duration of RA-TPE seems even shorter, probably because of the differences in the procedures, such as the necessity for dissection between the rectum and prostate in the narrow pelvis. Uehara et al. reported the feasibility of laparoscopic TPE compared to that of conventional TPE [6]. The authors mentioned that laparoscopic TPE should be applied for carefully selected patients. In the field of rectal cancer, particularly in men, because laparoscopic surgery is performed deeper in the pelvis, the range of the rigid forceps motion becomes further limited, and the dissection becomes more difficult. However, RA-TPE seems advantageous over laparoscopic surgery because of the increased dexterity of EndoWrist^®^ instruments, which provide a greater range of motion even in the narrow pelvis.

**Table 2:** Comparison of the outcomes between the reported RA-TPE cases and the other approaches for multivisceral exenteration in pelvis

Variables	RA-TPE of the reported cases for advanced rectal cancer requiring en bloc resection [12–15]	TPE against colorectal cancer [19]	Simultaneous RA-surgery for synchronous primary rectal and prostate cancer [20]	Laparoscopic TPE against pelvic malignancies invading adjacent organs [6]
Sample size	5	749	5	9
Age (year)				
Median (range)	61 (41–67)	59 (51.0–67.0)	72 (61–75)	64 (20–72))
Sex (male/female)	5/0	397/352	NA	5/4
BMI (kg/m^2^)				
Median (range)	19.0 (18.0–32.0)	25.1 (IQR; 22.0–29.6)	23.2 (20.8–24.8)	21.5 (19.0–31.0)
Disease				
Primary CRC	5	714	5	2
Locally recurrent rectal cancer	0	0	0	4
Other diseases	0	Anal cancer 33	0	3
Neoadjuvant treatment				
None	0	NA	4	3
Chemotherapy	5	NA	1	6
Radiotherapy	5	NA	0	0
Operative results				
Procedure				
TPE	5	749	0	9
Not TPE	0	0	5 (ISR + RP 2, APR + RP 2, AR + RP 1)	0
Operative duration (min)				
Median (range)	480 (200–570)	462 (333–582)	629 (431–764)	935 (716–1219)
Amount of blood loss				
Median (range)	250 (100–350, one missing)	NA (Transfusion rate; 50%)	100 (20–345)	830 (283–5225)
Conversion to the open surgery	0	–	0	1
R0 resection	5	NA	5 (as for rectal cancer)	7
Any complication	UTI 2, ileus 1, unknown fever 1	367	NA	UTI 4, ileus 4, wound infection 1
Major complication (Clavien–Dindo classification ≥ 3)	Ureteric stricture 1	120	Vesicourethral anastomotic leak 2, anastomotic leak 1	0
Postoperative hospital stay				
Median (range)	9 (7–21)	9 (IQR; 7–13)^†^	23 (11–27)	27 (23–53)

RA, robot-assisted; TPE, total pelvic exenteration; NA, not available; BMI, body mass index; IQR, interquartile range; CRC, colorectal cancer; ISR, intersphincteric resection; RP, radical prostatectomy; APR, abdominoperineal resection; AR, anterior resection; UTI, urinary tract infection

^†^including not only the patients with colorectal cancer but also urologic, gynecologic, and other malignancies

Our study provides considerable short-term outcomes of RA-TPE for primary advanced rectal cancer. Because of the small number of reported cases, the optimal criteria for RA-TPE have not been elucidated; therefore, its application should be limited to carefully selected patients. RA-TPE warrants further studies with more cases to estimate its exact feasibility.

## Conclusions

RA-TPE was performed for a patient with advanced rectal cancer without serious complications. RA-TPE may provide the advantages of minimally invasive surgery, particularly in the enclosed space of the pelvis.

## Data Availability

The data supporting the findings of this report are available from the corresponding author upon reasonable request.

## Abbreviations

TPE: Total pelvic exenteration
RA: Robot-assisted
CRT: Chemoradiotherapy
UTI: Urinary tract infection
